# Clinical relevance of proteinuria selectivity index and fractional excretion of sodium in patients with nephrotic syndrome

**DOI:** 10.1038/s41598-024-75281-9

**Published:** 2024-10-10

**Authors:** Takashin Nakayama, Tatsuhiko Azegami, Shintaro Yamaguchi, Keita Hirano, Motoaki Komatsu, Kentaro Fujii, Koji Futatsugi, Hidenori Urai, Takahisa Kawaguchi, Tomoaki Itoh, Norifumi Yoshimoto, Aika Hagiwara, Akihito Hishikawa, Hiroto Matsuda, Takashi Ando, Yasuyoshi Yamaji, Marohito Murakami, Akinori Hashiguchi, Yuko Kaneko, Takashi Yokoo, Kaori Hayashi

**Affiliations:** 1 Division of Nephrology, Endocrinology and Metabolism, Department of Internal Medicine, Keio University School of Medicine, 35 Shinanomachi, Shinjuku-Ku, Tokyo, 160-8582 Japan; 2 Division of Nephrology and Hypertension, Department of Internal Medicine, Jikei University School of Medicine, Tokyo, Japan; 3 Division of Nephrology, Department of Internal Medicine, Japanese Red Cross Ashikaga Hospital, Tochigi, Japan; 4 Department of Nephrology, Tokyo Saiseikai Central Hospital, Tokyo, Japan; 5 Department of Nephrology, Keiyu Hospital, Kanagawa, Japan; 6 Department of Nephrology, Tachikawa Hospital, Tokyo, Japan; 7 Division of Endocrinology, Metabolism and Nephrology, Department of Internal Medicine, Sano Kosei General Hospital, Tochigi, Japan; 8 Department of Nephrology, Kawasaki Municipal Hospital, Kanagawa, Japan; 9 Department of Nephrology, JCHO Saitama Medical Center, Saitama, Japan; 10 Department of Pathology, Keio University School of Medicine, Tokyo, Japan; 11 Division of Rheumatology, Department of Internal Medicine, Keio University School of Medicine, Tokyo, Japan

**Keywords:** Nephrotic syndrome, Proteinuria selectivity index, Fractional excretion of sodium, Complete remission, Minimal change disease, Nephrology, Kidney diseases

## Abstract

Proteinuria selectivity index (PSI) is a potential tool for histological classification and prediction of treatment response in nephrotic syndrome, but evidence is insufficient. Clinical relevance of fractional excretion of sodium (FENa) in nephrotic syndrome remains largely unexplored. This multicenter retrospective study included patients with nephrotic syndrome who underwent kidney biopsy between January 2012 and June 2022. Optimal cutoffs for predicting complete remission based on PSI and FENa were determined using receiver operating characteristic curves. Patients were divided into two groups using these cutoffs and followed until complete remission. Of the 611 patients included, 177 had minimal change disease (MCD), 52 had focal segmental glomerulosclerosis (FSGS), and 149 had membranous nephropathy (MN). Median (interquartile range) PSI were 0.14 (0.09–0.19) for MCD, 0.33 (0.23–0.40) for FSGS, and 0.20 (0.14–0.30) for MN. FENa were 0.24 (0.09–0.68), 1.03 (0.50–2.14), and 0.78 (0.41–1.28). Patients with low PSI and FENa had a higher incidence of complete remission. Cox regression analyses demonstrated that both parameters were associated with achieving complete remission (HR 2.73 [95% CI 1.97–3.81] and HR 1.93 [95% CI 1.46–2.55], respectively). PSI and FENa may be useful for histological classification and predicting remission in nephrotic syndrome.

## Introduction

Nephrotic syndrome, characterized by severe proteinuria and hypoalbuminemia, is associated with an increased risk of life-threatening complications or end-stage kidney disease^1–3^. Individuals of any age can develop nephrotic syndrome, with generation-specific variations in histological types. Epidemiological data in adults are limited; the incidence was previously reported to be approximately three cases per 10,000 patient-years, although a recent Danish study suggested the possibility of significant underestimation^4,5^.

The concept of proteinuria selectivity was proposed in the 1960s to reflect changes in the glomerular permeability. It has since been recognized as a potential method of predicting treatment responses and classifying the histological types of nephrotic syndrome^6–15^. However, because most studies evaluating this parameter have used a single-centered design with small sample size, the evidence is limited. Indeed, KDIGO clinical practice guidelines contain little information on proteinuria selectivity and it is not widely assessed anywhere in the world^16^. Consequently, it is important to verify the significance of proteinuria selectivity in larger datasets.

Fractional excretion of sodium (FENa) may also have relevance in nephrotic syndrome. Although patients with nephrotic syndrome commonly present with edema, it is noteworthy that the underlying mechanisms and the extent of intravascular volume vary according to histologic patterns^17–19^. Given that FENa decreases along with effective circulating volume, it may also be a valuable tool in clinical practice^20,21^.

Herein, we conducted a multicenter study to comprehensively investigate the distribution of proteinuria selectivity index (PSI) and FENa across the different histologic types of nephrotic syndrome. Furthermore, we used sophisticated statistical approaches to evaluate whether these parameters could predict the achievement of complete remission of nephrotic syndrome.

## Results

### Patients

Of 718 patients with nephrotic syndrome who underwent kidney biopsy, 107 were excluded (66 with histological types other than those specified, three with biopsies that could not be interpreted, and 38 without data on both PSI and FENa). A total of 611 patients were included in the present study (Supplementary Fig. 1). The histological types of nephrotic syndrome in the study population were minimal change disease (MCD) (n = 177), focal segmental glomerulosclerosis (FSGS) (n = 52), membranous nephropathy (MN) (n = 149), membranoproliferative glomerulonephritis (MPGN) (n = 27), immunoglobulin A nephropathy (IgAN) (n = 51), lupus nephritis (LN) (n = 33), renal amyloidosis (RA) (n = 33), and diabetic kidney disease (DKD) (n = 89).

### PSI and FENa according to histological type

**Fig. 1:**
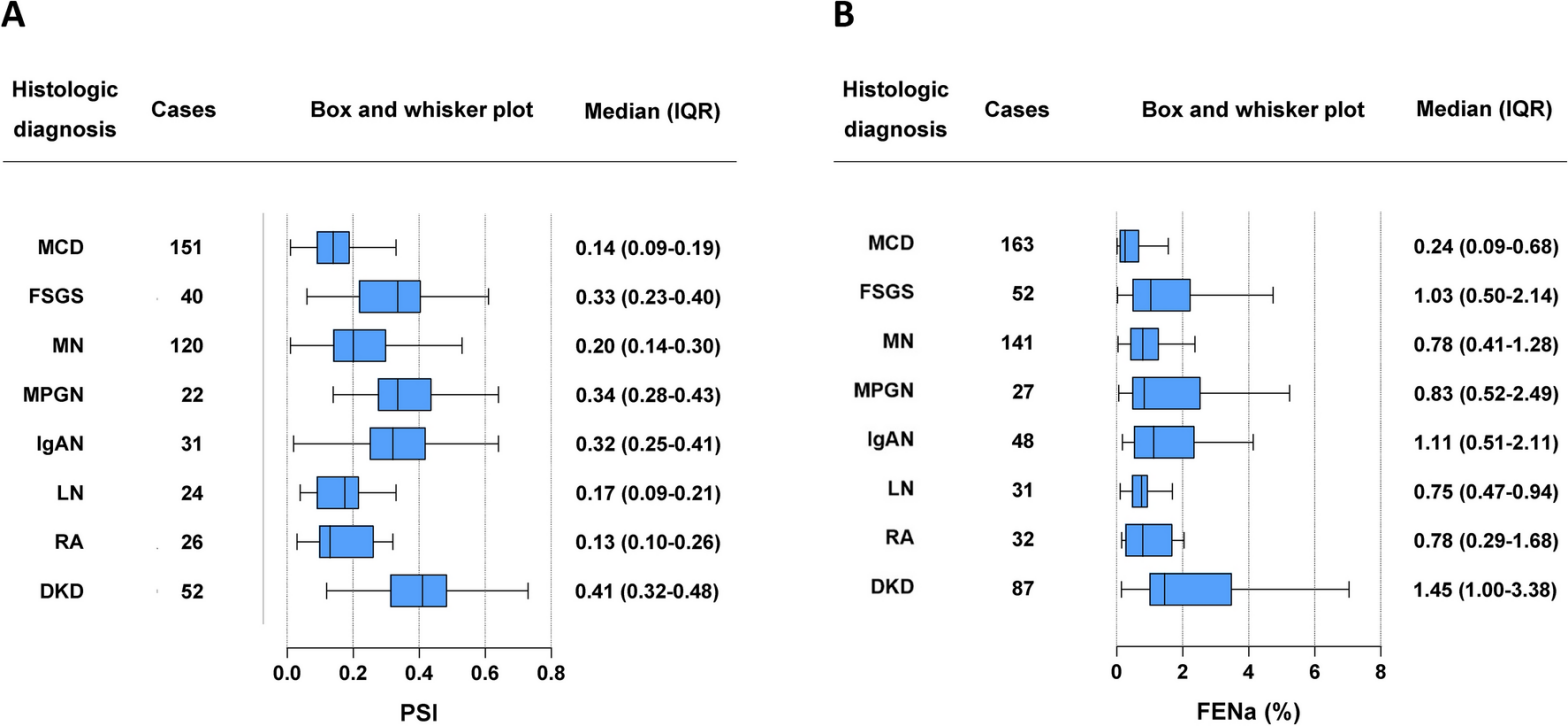
PSI and FENa by histological classification. The distribution of (**a**) PSI and (**b**) FENa across histological types is presented. Box plots show the IQR with a line at the median, and whiskers extend to 1.5 times the IQR. *IQR* interquartile range, *PSI* proteinuria selectivity index, *FENa* fractional excretion of sodium.

The PSI and FENa values are shown for each histological type (Fig. 1). Median PSI was low in patients with MCD (0.14 [0.09*–*0.19]), RA (0.13 [0.10*–*0.26)], and LN (0.17 [0.09*–*0.21]), followed by MN (0.20 [0.14*–*0.30]). In contrast, patients with DKD had an elevated PSI (0.41 (0.32*–*0.48]). Median FENa was low in patients with MCD (0.24 [0.09*–*0.68]) and high in those with DKD (1.45 [1.00*–*3.38]).

In the dataset after excluding patients with RA and DKD, a moderate correlation was observed between FENa and PSI (Rho = 0.35, P < 0.001) (Supplementary Fig. 2a). The area under the receiver operating characteristic curve was 0.75 (0.70*–*0.80) for PSI and 0.69 (0.64*–*0.74) for FENa (Supplementary Fig. 2b). The optimal thresholds for predicting complete remission based on PSI and FENa were 0.20 and 0.65, respectively. Patients were then divided into two groups per parameter based on these thresholds. In the low-PSI (≤ 0.20) and low-FENa (≤ 0.65) groups, patients were significantly younger and had significantly lower systolic blood pressure, less comorbid diabetes and hypertension, lower serum albumin and total protein, higher estimated glomerular filtration rate (eGFR), lower proportions of global glomerulosclerosis, and less severe interstitial fibrosis and tubular atrophy (IFTA) compared with those who had higher PSI and FENa (Table 1). Red blood cell (RBC) counts were significantly lower in the low-PSI group, but not in the low-FENa group. Conversely, the opposite trend was observed in terms of urinary protein levels. The low-PSI and low-FENa groups showed a tendency to receive corticosteroid. The majority of patients with MCD had lower values of these indicators (Supplementary Fig. 3).

**Table 1:** Characteristics of study patients stratified by PSI and FENa cutoff values.

Variable	PSI (n = 388)			FENa (n = 462)		
	Low (n = 205)	High (n = 183)	P value	Low (n = 240)	High (n = 222)	P value
Age, year	61 (42–73)	69 (54–77)	< 0.001	62 (44–73)	71 (54–78)	< 0.001
Female, n (%)	94 (45.9)	74 (40.4)	0.33	101 (42.1)	95 (42.8)	0.95
Body mass index, kg/m^2^	23.3 (20.9–26.5)	24.0 (21.3–26.5)	0.49	23.6 (21.1–26.9)	23.0 (20.9–25.5)	0.07
Blood pressure, mmHg						
Systolic	125 (114–138)	138 (126–155)	< 0.001	130 (115–146)	132 (123–150)	0.02
Diastolic	76 (70–84)	81 (68–90)	0.10	78 (69–88)	77 (69–84)	0.20
Use of diuretics, n (%)	122 (59.5)	112 (61.2)	0.81	138 (57.5)	128 (57.7)	1.00
Comorbidities, n (%)						
Hypertension	78 (38.0)	106 (57.9)	< 0.001	96 (40.0)	129 (58.1)	< 0.001
Diabetes	23 (11.2)	40 (21.9)	0.007	36 (15.0)	45 (20.3)	0.17
Blood test						
Albumin, g/dl	1.7 (1.3–2.3)	2.1 (1.6–2.7)	< 0.001	1.8 (1.3–2.5)	2.3 (1.7–2.8)	< 0.001
Protein, g/dl	4.8 (4.2–5.4)	5.3 (4.6–5.9)	< 0.001	4.8 (4.2–5.7)	5.3 (4.6–5.9)	< 0.001
Creatinine, mg/dl	0.86 (0.69–1.05)	1.35 (0.96–2.02)	< 0.001	0.94 (0.72–1.30)	1.12 (0.82–2.02)	< 0.001
eGFR, ml/min/1.73 m2	65.7 (51.7–79.1)	39.2 (23.5–56.0)	< 0.001	58.8 (40.8–75.4)	43.5 (24.4–65.6)	< 0.001
Urine test						
Urinary protein, g/gCr	7.8 (5.2–11.1)	7.6 (5.1–11.2)	0.98	8.2 (4.9–11.6)	6.5 (4.7–9.2)	0.004
Urinary RBC, grade^†^	0 (0–1)	1 (0–3)	< 0.001	1 (0–3)	1 (0–2)	0.79
Kidney biopsy						
Global sclerosis ratio, %	6.7 (0.0–16.7)	20.0 (6.1–38.8)	< 0.001	9.1 (0.0–21.4)	18.2 (6.3–40.0)	< 0.001
IFTA, grade*	0 (0–1)	1 (0–2)	< 0.001	0 (0–1)	1 (0–2)	< 0.001
Immunosuppressants, n (%)						
Corticosteroid	182 (88.8)	145 (79.2)	0.02	204 (85.0)	174 (78.4)	0.09
Others	70 (34.1)	76 (41.5)	0.16	92 (38.3)	83 (37.4)	0.91

*eGFR* estimated glomerular filtration rate, *FENa* fractional excretion of sodium, *IFTA* interstitial fibrosis and tubular atrophy, *MCD* minimal change disease, *PSI* proteinuria selectivity index, *RBC* red blood cell.

^†^Five grades based on number of cells per high power field: Grade 1 (≤ 5), Grade 2 (6–10), Grade 3 (11–25), Grade 4 (26–50), and Grade 5 (≥ 51).

*Four grades: Grade 0 (< 10%), Grade 1 (10%–25%), Grade 2 (26%–50%), and Grade 3 (> 50%).

### Predictive value of PSI and FENa for remission

**Fig. 2:**
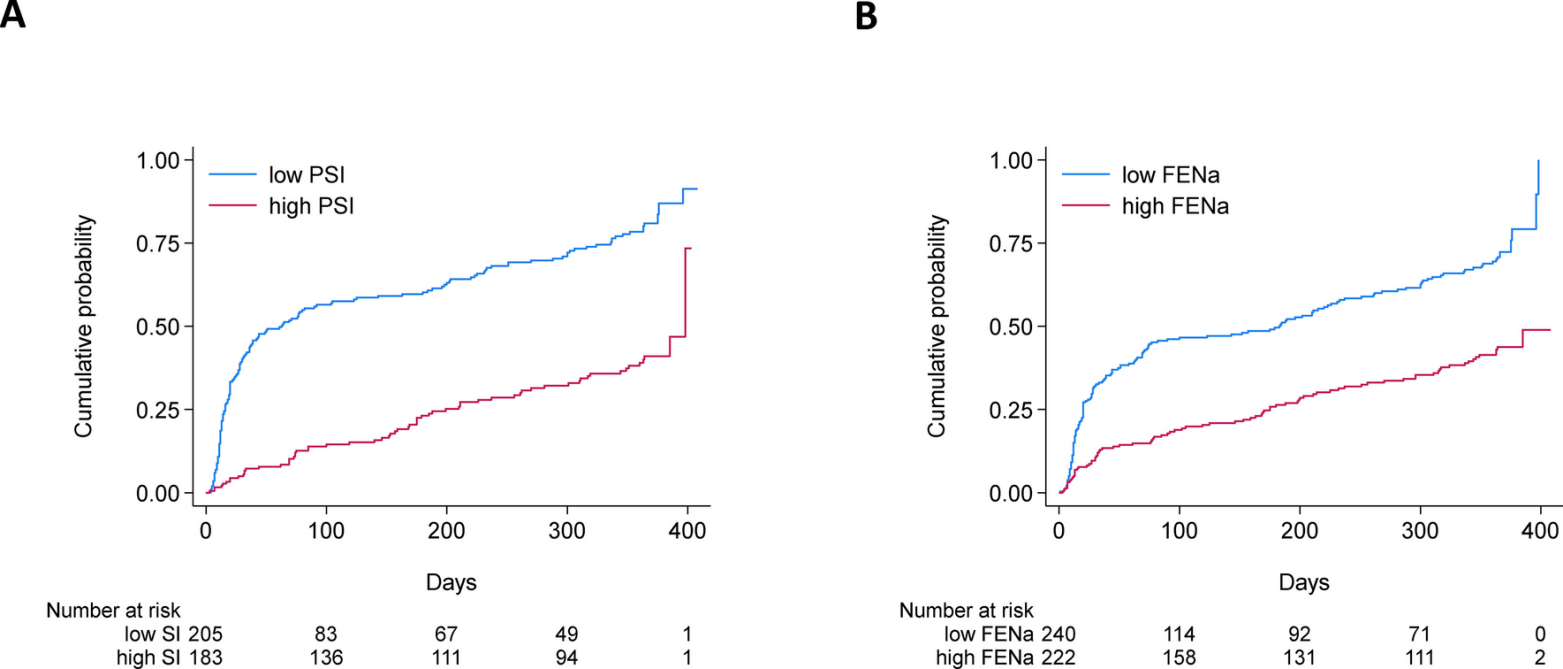
Kaplan–Meier curves for complete remission. Patients were divided into two groups based on (**a**) PSI and (**b**) FENa cutoffs. The Kaplan–Meier plots show the cumulative incidence of complete remission (log-rank P < 0.001 for both parameters). *PSI* proteinuria selectivity index, *FENa* fractional excretion of sodium.

During a median follow-up of 175 (31*–*363) days, 158 of 205 patients (77.1%) in the low-PSI group and 64 of 183 (35.0%) in the high-PSI group reached complete remission. The Kaplan–Meier curves and the log-rank test indicated a significantly higher cumulative incidence of complete remission in the low-PSI group (P < 0.001) (Fig. 2a). Complete remission was observed in 159 of 240 patients (66.2%) with low FENa and 84 of 222 patients (37.8%) with high FENa; the cumulative incidence was significantly higher in the low-FENa group (P < 0.001) (Fig. 2b). In the overall population, unadjusted Cox regression analysis showed that low PSI was associated with a higher incidence of complete remission (Table 2); this result remained consistent in Cox regression analysis adjusted for age, sex, and body mass index (BMI). Multivariate Cox regression analysis (Model 3) also demonstrated that low PSI independently predicted the achievement of complete remission; the hazard ratio (HR) was 2.73 (95% confidence intervals [CI], 1.97*–*3.81). In a Cox model to evaluate FENa, unadjusted analysis showed a significant association between its low value and an elevated rate of complete remission. Even after adjusting for confounders (Model 3), FENa remained associated with complete remission (HR 1.93 [95% CI 1.46*–*2.55]). The concordance index for the models assessing PSI and FENa was 0.76 (0.73*–*0.80) and 0.75 (0.71*–*0.78), respectively. The results of subgroup analysis showed an association of PSI with complete remission in both MCD and non-MCD groups (HR 2.59 [95% CI 1.47–4.56] and HR 1.81 [95% CI 1.14*–*2.89], respectively); the HRs for FENa were 1.29 (95% CI 0.85*–*1.97) and 1.57 (95% CI 1.04*–*2.36) in these respective groups.

**Table 2:** Results of Cox regression analyses for complete remission.

	PSI		FENa	
	HR (95%CI)	P value	HR (95%CI)	P value
Overall				
Model 1	3.58 (2.67–4.79)	< 0.001	2.40 (1.84–3.13)	< 0.001
Model 2	3.51 (2.60–4.74)	< 0.001	2.19 (1.67–2.87)	< 0.001
Model 3	2.73 (1.97–3.81)	< 0.001	1.93 (1.46–2.55)	< 0.001
MCD				
Model 1	3.05 (1.85–5.05)	< 0.001	1.28 (0.86–1.91)	0.22
Model 2	2.96 (1.75–4.99)	< 0.001	1.14 (0.75–1.74)	0.53
Model 3	2.59 (1.47–4.56)	0.001	1.29 (0.85–1.97)	0.23
non-MCD				
Model 1	2.10 (1.40–3.14)	< 0.001	1.99 (1.37–2.90)	< 0.001
Model 2	2.11 (1.40–3.19)	< 0.001	1.88 (1.28–2.75)	0.001
Model 3	1.81 (1.14–2.89)	0.01	1.57 (1.04–2.36)	0.03

Model 1: unadjusted; Model 2: age, sex, and body mass index; Model 3: Model 2 plus hypertension, diabetes, estimated glomerular filtration rate, urinary protein, and urinary red blood cell count.

*CI* confidence interval, *FENa* fractional excretion of sodium, *HR* hazard ratio, *MCD* minimal change disease, *PSI* proteinuria selectivity index.

### Sensitivity analyses

We conducted six sensitivity analyses. First, in a Cox multivariate analysis replacing eGFR with the proportion of global glomerulosclerosis as a covariate, both low PSI and FENa were associated with reaching complete remission (HR 2.49 [95% CI 1.81*–*3.44] and HR 1.85 [95% CI 1.40*–*2.44], respectively) (Supplementary Table 1). Second, multivariate analyses using IFTA as a covariate consistently showed that PSI and FENa were predictors of complete remission (HR, 2.35 [95% CI 1.68*–*3.27] and HR 1.89 [95% CI 1.43*–*2.50], respectively) (Supplementary Table 2). Third, we defined partial remission as the outcome, and both low PSI and FENa were associated with an increased incidence (HR 2.31 [95% CI 1.73*–*3.08] and HR 1.59 [95% CI 1.25*–*2.04], respectively) (Supplementary Table 3). Fourth, we excluded 184 patients who did not receive immunosuppressive agents; our primary findings remained consistent (HR 2.89 [95% CI 2.05*–*4.09] and HR 1.97 [95% CI 1.47*–*2.64], respectively) (Supplementary Table 4). Fifth, we performed an analysis considering a competing risk, revealing that both PSI and FENa predicted subsequent complete remission (HR 2.83 [95% CI 2.02*–*3.96] and HR 1.93 [95% CI 1.47*–*2.54], respectively) (Supplementary Fig. 4) (Supplementary Table 5). Sixth, similar results were observed using a multivariate Cox regression model with multiple imputations for missing values (HR 2.86 [95% CI 2.06*–*3.98] and HR 1.86 [95% CI 1.41*–*2.46], respectively) (Supplementary Table 6).

## Discussion

This multicenter study investigated the distribution of PSI across histological type of nephrotic syndrome and validated its ability to predict remission in patients. The value of PSI exhibited distinct characteristics across different histological classification, while noticeable overlap was also observed. A multitude of analyses demonstrated that low PSI is significantly associated with an increased remission rate, and this finding remained consistent even when analyzing MCD and non-MCD patients separately. At the same time, we investigated the clinical implication of baseline FENa in nephrotic syndrome and found that this parameter could be somewhat predictive of remission.

The significance of urinary protein selectivity in nephrotic syndrome dates back to the 1960s when Cameron et al. demonstrated a potential relationship between PSI and steroid responsiveness^8^. Subsequently, studies by Laurent et al. (1993), Bazzi et al. (2000), and Nakamura et al. (2019) reported that PSI was lower in patients with MCD than in those with other histologic types, and that lower PSI might indicate a better response to treatment. Each study included 52 patients (39 MCD and 13 others), 89 patients (9 MCD, 29 FSGS, and 51 MN), and 49 patients (20 MCD, 4 FSGS, 13 MN, and 12 others), respectively^11,13,14^. In other studies, Mallick et al. demonstrated that 9 of 12 patients with MN who achieved remission had low PSI, compared to only 8 of the remaining 28 patients with sustained proteinuria^9^. From these observations, PSI could be a promising tool in nephrotic syndrome management. However, these studies were modest in scale and examined a limited range of diseases. Analyses to evaluate the predictive ability of PSI did not incorporate robust statistical techniques; potential confounders such as comorbid conditions or histological findings were not considered. Thus, the utility of PSI as a prognostic indicator was not fully established.

Dysfunctional slit diaphragms in the glomerular capillary wall, composed of numerous small pores with a few large pores and shunts, are involved in the pathogenesis of nephrotic syndrome^22,23^. With the loss of negative charge, only small-molecular-weight proteins like transferrin can efficiently pass through the glomerular barrier via small pores; high-molecular-weight proteins like immunoglobulin G (IgG) cannot, resulting in highly selective proteinuria. Conversely, an increase in the number of large pores and shunts caused by structural changes in the glomerular capillary wall may lead to leakage of proteins of any molecular weight into urine, diminishing the selectivity of urinary protein. Theoretically, the presence of low selective proteinuria may suggest a more severe pathological condition.

The present study expanded the evidence on PSI in nephrotic syndrome using collaborative data from multiple institutions. Our comprehensive investigation confirmed that patients with MCD had markedly low PSI values, as previously reported^14^. Trends towards higher PSI in MN and further elevation in FSGS were generally consistent with prior findings^13^. This could imply that structural changes in the glomerular capillary wall are subtle in MCD, while more pronounced in FSGS. We obtained novel insights into proteinuria selectivity across MPGN, IgAN, DKD, LN, and RA; the first three nephrotic conditions and the last two showed PSI values similar to FSGS and MN, respectively. On the other hand, PSI showed a widespread distribution within each histological type. The limited availability of clinical data makes it challenging to elucidate the reasons behind this phenomenon; nevertheless, factors such as differences in the severity of renal dysfunction (glomerular sclerosis and IFTA) based on the characteristics of each histological type and variations in the disease duration at the time of biopsy may have an impact. Additionally, we conducted multivariate Cox regression adjusted for various confounders, revealing an association between low PSI and an increased likelihood of complete remission. This association was observed in subgroups of both MCD and non-MCD patients, and six sensitivity analyses consistently supported our main results. Although the exact mechanism remains unclear, these findings could derive from a potential association between proteinuria selectivity and structural changes in the glomerular barrier.

We also examined the significance of FENa in nephrotic syndrome. Two mechanisms have been hypothesized to explain the interstitial fluid increase in nephrotic syndrome: the underfill mechanism, which involves secondary sodium reabsorption due to intravascular volume depletion, and the overfill mechanism, which involves primary sodium retention leading to intravascular volume expansion^17^. The dominance of these mechanisms varies according to case and histological type^18,19^. Even in the nephrotic state, FENa typically decreases with intravascular contraction^20,21^. Our finding that the patients with MCD tended to have low FENa appear to be compatible with its predominant underfill mechanism. The observed correlation between FENa and PSI may be attributable to each having its substantially low value in MCD. Surprisingly, a range of analyses demonstrated that low FENa was associated with an increased probability of complete remission, although its predictive performance was somewhat inferior to PSI. These findings imply that patients with nephrotic syndrome who are susceptible to the underfill mechanism, could potentially have a favorable prognosis. This is an intriguing area for future research.

The present study has clinical implications. There is no doubt about the importance of kidney biopsy in the clinical care of nephrotic syndrome. However, concerns about sampling bias remain with kidney biopsy; in particular, when the number of glomeruli obtained by sampling is limited, it becomes challenging to accurately differentiate between MCD and FSGS, as well as to assess the effects of conditions such as hypertension and diabetes. Furthermore, depending on the patient’s condition, performing a biopsy may be challenging. Admittedly, PSI and FENa values overlap across histological types; nevertheless, their low cost, non-invasive nature, and rapid results might offer a certain degree of value in differentiating histological types. On the other hand, the evaluation of these indicators appears to hold significant value in terms of the predictive ability. While histological types provide valuable information for predicting treatment response, adding PSI and FENa might offer higher accuracy. Our findings confirm the robust relationship between remission and these indicators in both MCD and non-MCD patients. This complementary aspect may contribute to refining the management of nephrotic syndrome.

The strength of our current study includes the broad recruitment of patients with biopsy-confirmed nephrotic syndrome from hospitals of varying sizes, ranging from university hospitals to community-based settings. This approach allowed us to extensively investigate PSI and FENa across different histological types and conduct a detailed analysis of their potential for predicting remission. Consequently, we believe that our findings have satisfactory external validity. However, this study has several limitations. First, it was a retrospective cohort study. Despite adjusting numerous variables in the multivariate models, the possibility of unmeasured confounders still exits; factors such as the time from onset to treatment, the presence of anti-PLA2R antibodies in MN patients, and the subclasses of FSGS (primary, genetic, and secondary forms). Also, concerns about information bias persist. Second, PSI and FENa were measured only once at the time of kidney biopsy. The stability of these parameters in nephrotic conditions has not been confirmed, necessitating caution in interpreting the results. Also, fluctuations in these values during treatment and around the time of relapse were not evaluated. Third, we defined PSI as the clearance ratio of IgG to transferrin. The utility of proteinuria selectivity calculated using alternative methods, such as IgG to albumin, remains unvalidated, although significant correlations between these indices have been reported^10^. Fourth, the area under the receiver operating characteristic curve was not sufficiently high; 0.75 (0.70–0.80) for PSI and 0.69 (0.64–0.74) for FENa. Lastly, this study did not evaluate associations between the values of PSI and FENa and the subsequent kidney function trajectory. Future studies are needed to clarify these issues.

In conclusion, this study has established the distinct distribution patterns of PSI across histological types and demonstrated the predictive value of PSI for nephrotic syndrome remission. Proactively measuring this non-invasive indicator may offer benefits for patients with nephrotic syndrome. Furthermore, FENa also showed some predictive ability; given its cost-effectiveness and the availability of rapid test results, FENa assessment may also be worth considering.

## Methods

### Study participants

This retrospective study was conducted at Keio University Hospital and seven other hospitals in Japan. We included patients aged ≥ 18 years with nephrotic syndrome who received a native kidney biopsy between January 2012 and June 2022. Nephrotic syndrome was defined as the presence of both severe proteinuria (either 24-h urine collection ≥ 3.5 g/day or spot urine protein-to-creatinine ratio ≥ 3.5 g/gCr) and hypoalbuminemia (serum albumin levels ≤ 3.0 g/dL)^24^. The histological classification of nephrotic syndrome included MCD, FSGS, MN, MPGN, IgAN, LN, RA, and DKD. The exclusion criteria were as follows: 1) patients with histological types other than those specified above; 2) patients without a definitive diagnosis due to insufficient samples; 3) patients lacking both PSI and FENa data. Using data from all participants included, we analyzed the distribution of PSI and FENa across different histological classifications. Next, we created a dataset excluding RA and DKD to evaluate the impact of PSI and FENa on treatment response. This study and all its protocols were reviewed and approved by the Keio University School of Medicine Ethics Committee (approval number: 20231146), and adhered to the tenets of the Declaration of Helsinki. Informed consent was obtained by providing patients with an opt-out on the website.

### Data collection

Demographic data including age, sex, height, weight, BMI, blood pressure, use of diuretics and comorbidities at the time of kidney biopsy were collected through electronic medical record review. The following laboratory information at the point closest to the biopsy before treatment initiation was also obtained: serum albumin levels, serum and urinary levels of protein, creatinine, electrolytes, transferrin, and IgG, and urinary RBC counts. eGFR was calculated using the following equation: 194 × Cr^-1.094^ × Age^-0.287^ (× 0.739 if female)^25^. PSI was determined using the clearance ratio of IgG to transferrin: (urinary IgG × serum transferrin) / (serum IgG × urinary transferrin)^15^. FENa was calculated using the formula: (urinary sodium × serum creatinine) / (urinary creatinine × serum sodium) × 100^26^. Urinary RBC counts were classified into five grades of severity based on the number of cells per high power field: Grade 1 (≤ 5), Grade 2 (6–10), Grade 3 (11–25), Grade 4 (26–50), and Grade 5 (≥ 51). Regarding the kidney biopsy, data on the histological classification, the total number of glomeruli, the number of globally sclerosed glomeruli, and the severity of IFTA were collected. The proportion of global glomerulosclerosis was calculated, and IFTA was categorized into four groups: Grade 0 (< 10%), Grade 1 (10%–25%), Grade 2 (26%–50%), and Grade 3 (> 50%)^27^.

### Follow-up

The assessment of therapeutic efficacy in nephrotic syndrome was based on the amount of proteinuria; complete remission was defined as urine protein < 0.3 g/gCr and partial remission as ≥ 0.3 g/gCr to < 1.0 g/gCr^24^. Study participants were followed up from kidney biopsy until complete remission, initiation of maintenance dialysis, or death, or until the end of the observation period (1 year after kidney biopsy). Additionally, any immunosuppressive agents used to treat nephrotic syndrome were documented.

### Statistical analyses

The clinical characteristics of study participants in the dataset for analysis of the predictive ability of PSI and FENa for remission were stratified into two groups based on the cutoff values of PSI and FENa, and then summarized for each group. Continuous variables were expressed as median (25th–75th percentiles), and categorical variables as numbers (percentages). Differences between two continuous variables were analyzed using the Mann–Whitney U-test. The Chi-square test was employed to calculate the statistical significance of differences between categorical variables.

The relationship between PSI and FENa was assessed using the Spearman rank correlation coefficient. The ability of these parameters to predict complete remission was evaluated using receiver operating characteristic curve analyses, and the Youden index provided the optimal cutoff for each. Survival curves were visualized using the Kaplan–Meier method and compared using the log-rank test. From the perspective of assessing PSI values in relation to nephrotic syndrome remission, based on previous reports^13^, assuming a remission rate of 29% for nephrotic syndrome with low PSI, a hazard ratio of 2.0, a significance level (a) of 0.05, and a power (1-b) of 0.8, the required total sample size is estimated to be 112. Cox regression analyses were used to investigate the association between PSI or FENa and complete remission. HRs with 95% CIs were determined in three models: Model 1 (unadjusted), Model 2 (adjusted for age, sex, and BMI), and Model 3 (adjusted for hypertension, diabetes, eGFR, urinary protein levels, urinary RBC counts, and the variables included in Model 2). These covariates were selected based on clinical considerations regarding urinary protein excretion. Because MCD is distinguished by its favorable treatment response, we performed a subgroup analysis stratified by MCD or non-MCD.

To confirm the robustness of the findings, we performed six sensitivity analyses. First, we conducted a multivariate analysis selecting the proportion of global glomerulosclerosis instead of eGFR as a covariate. Second, we substituted IFTA for eGFR as an independent variable. Third, we redefined the outcome as the achievement of partial remission. Fourth, we included only those patients who received immunosuppressive therapy. Fifth, taking into account the potential competing risk of dialysis initiation and death, we employed the cumulative incidence function method and the subdistributional hazard model. Sixth, we used multivariate imputation by chained equations to deal with missing data (50 imputations); this imputation procedure considered all variables associated with the multivariate models.

A P-value of < 0.05 was considered to be statistically significant. All statistical analyses were performed using STATA version 18.

## Supplementary Information

Supplementary Figures.

Supplementary Tables.

## Data Availability

The data underlying this article will be shared on reasonable request to the corresponding author.
