# Supplementary material for: Clinical relevance of proteinuria selectivity index and fractional excretion of sodium in patients with nephrotic syndrome
Source: Sci Rep. 2024 Oct 10;14:23755. doi: 10.1038/s41598-024-75281-9 (PMC11467306; doi:10.1038/s41598-024-75281-9)
Supplement: Supplementary file 1 — Supplementary Figures. [file 41598_2024_75281_MOESM1_ESM.pdf]

Supplementary Figure. 1 Flowchart of study participants.

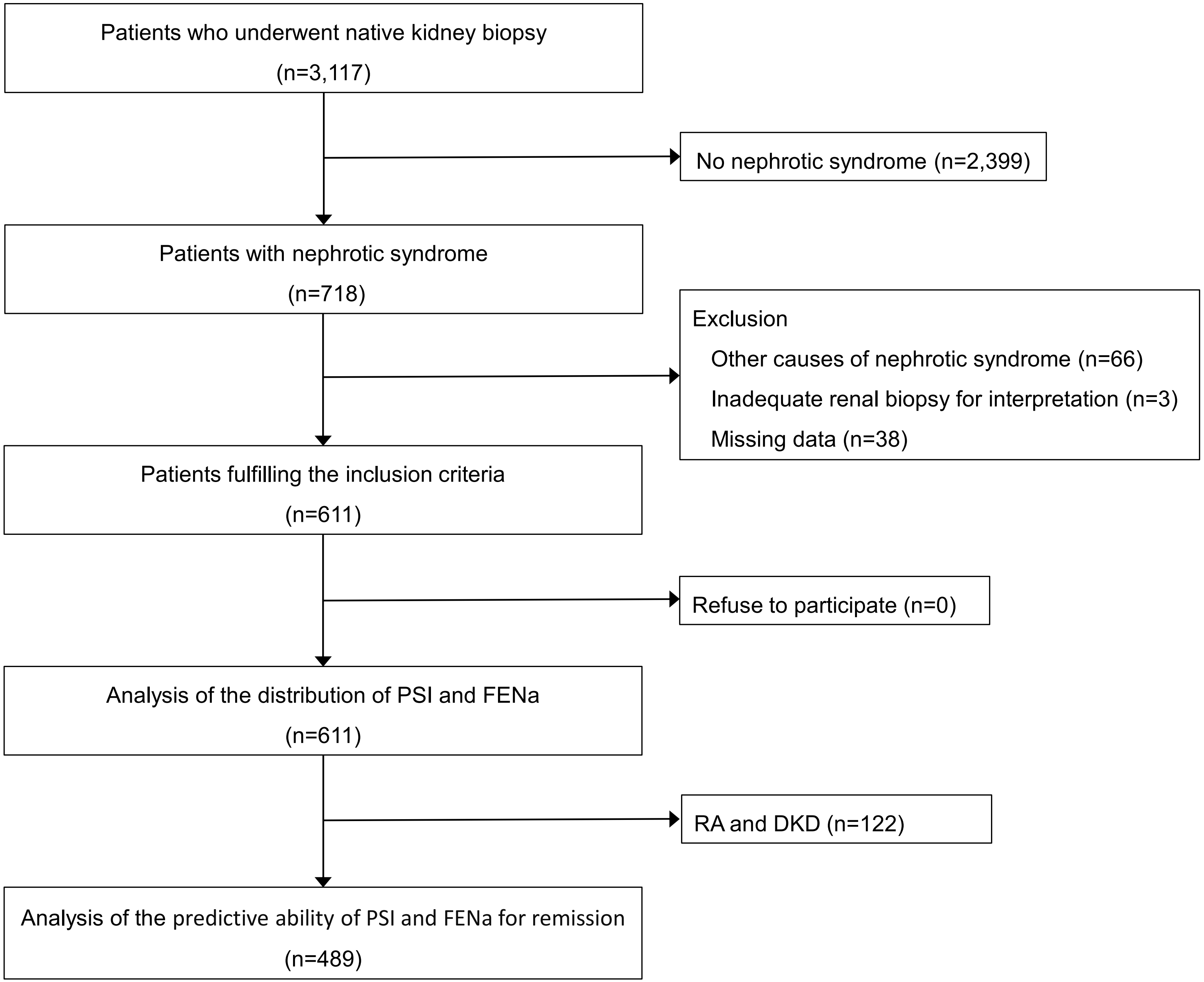

PSI, proteinuria selectivity index; FENa, fractional excretion of sodium; RA, renal amyloidosis; DKD, diabetic kidney disease.

Supplementary Figure. 2 Scatterplot and receiver operating characteristic curve.

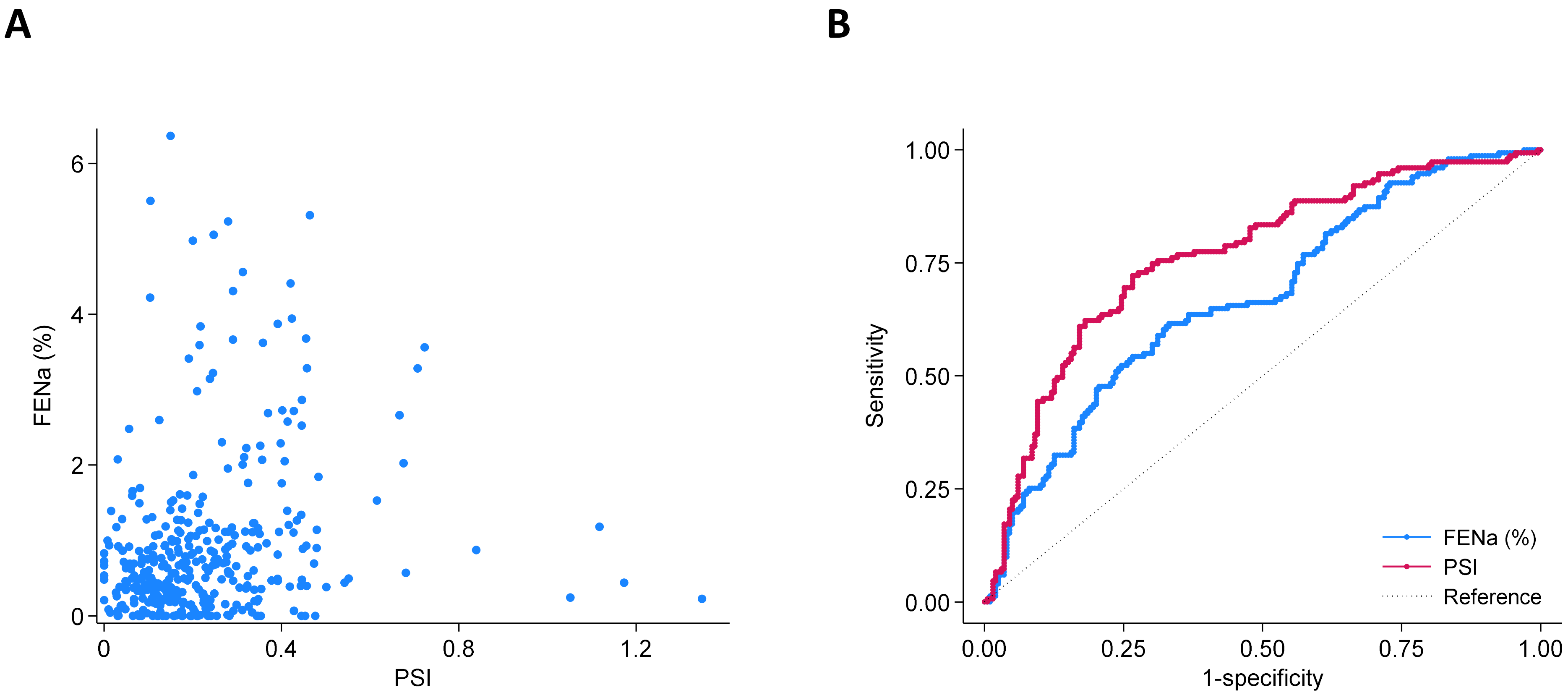

(A) Scatterplot showing a moderate correlation between PSI and FENa (Rho = 0.35, P < 0.001, Spearman correlation rank test).
(B) The receiver operating characteristic curve for achieving complete remission for PSI (0.75 [0.70–0.80]) and FENa (0.69 [0.64–0.74]).
Abbreviations: PSI, proteinuria selectivity index; FENa, fractional excretion of sodium.

Supplementary Figure. 3
Proportion of patients classified based on PSI and FENa cutoff values for each histological classification.

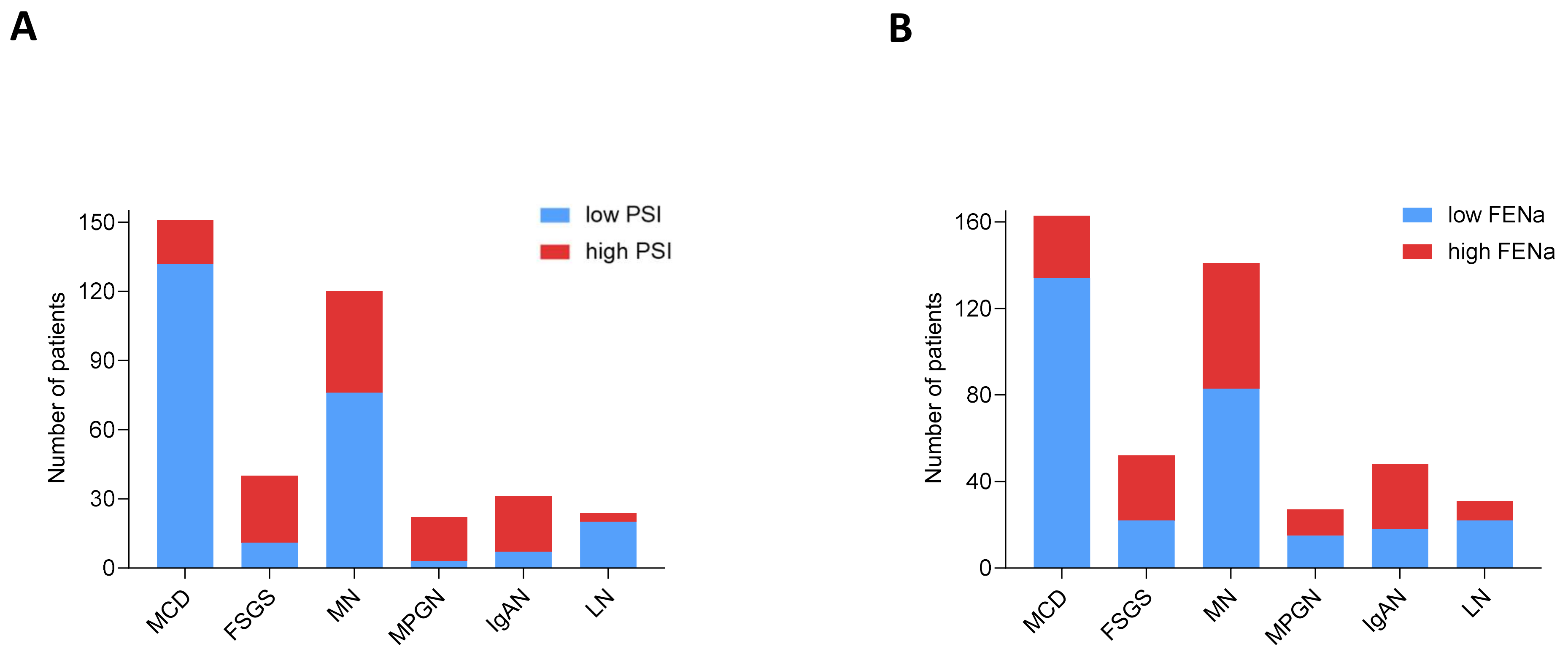

The proportions of low/high PSI (A) and FENa (B) are shown for each histological classification.

Abbreviations: DKD, diabetic kidney disease; FENa, fractional excretion of sodium; FSGS, focal segmental glomerulosclerosis; IgAN, immunoglobulin A nephropathy; LN, lupus nephritis; MCD, minimal change disease; MN, membranous nephropathy; MPGN, membranoproliferative glomerulonephritis; PSI, proteinuria selectivity index; RA, renal amyloidosis.

Supplementary Figure. 4
Competitive risk-adjusted cumulative incidence curves for complete remission.

A

B

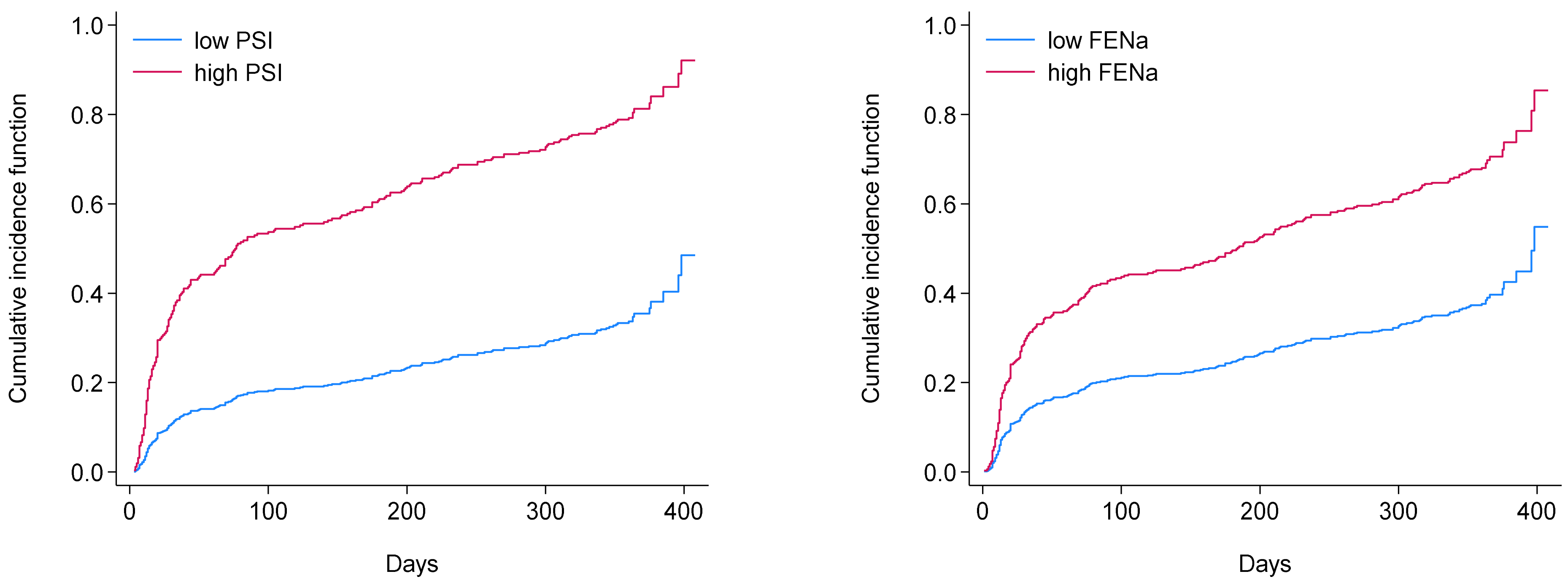

Patients were divided into two groups based on (A) PSI and (B) FENa cutoffs.

The cumulative incidence function method shows the cumulative incidences of complete remission. Abbreviations: PSI, proteinuria selectivity index; FENa, fractional excretion of sodium.
