# Supplementary material for: Clinical relevance of proteinuria selectivity index and fractional excretion of sodium in patients with nephrotic syndrome
Source: Sci Rep. 2024 Oct 10;14:23755. doi: 10.1038/s41598-024-75281-9 (PMC11467306; doi:10.1038/s41598-024-75281-9)
Supplement: Supplementary file 2 — Supplementary Tables. [file 41598_2024_75281_MOESM2_ESM.pdf]

Supplementary Table 1. Results of Cox regression analyses for complete remission (adjusted for proportion of global glomerulosclerosis instead of estimated glomerular filtration rate)

	PSI		FENa	
	HR (95%CI)	P value	HR (95%CI)	P value
Model 1	3.58 (2.67–4.79)	< 0.001	2.40 (1.84–3.13)	< 0.001
Model 2	3.51 (2.60–4.74)	< 0.001	2.19 (1.67–2.87)	< 0.001
Model 3	2.49 (1.81–3.44)	< 0.001	1.85 (1.40–2.44)	< 0.001

Model 1: unadjusted; Model 2: age, sex, and body mass index; Model 3: Model 2 plus hypertension, diabetes, proportion of global glomerulosclerosis, urinary protein, and urinary red blood cell. Abbreviations: CI, confidence interval; FENa, fractional excretion of sodium; HR, hazard ratio; PSI, proteinuria selectivity index.

Supplementary Table 2. Results of Cox regression analyses for complete remission (adjusted for interstitial fibrosis and tubular atrophy instead of estimated glomerular filtration rate)

	PSI		FENa	
	HR (95%CI)	P value	HR (95%CI)	P value
Model 1	3.58 (2.67–4.79)	< 0.001	2.40 (1.84–3.13)	< 0.001
Model 2	3.51 (2.60–4.74)	< 0.001	2.19 (1.67–2.87)	< 0.001
Model 3	2.35 (1.68–3.27)	< 0.001	1.89 (1.43–2.50)	< 0.001

Model 1: unadjusted; Model 2: age, sex, and body mass index; Model 3: Model 2 plus hypertension, diabetes, interstitial fibrosis and tubular atrophy, urinary protein, and urinary red blood cell count. Abbreviations: CI, confidence interval; FENa, fractional excretion of sodium; HR, hazard ratio; PSI, proteinuria selectivity index.

Supplementary Table 3. Results of Cox regression analyses for partial remission

	PSI		FENa	
	HR (95%CI)	P value	HR (95%CI)	P value
Model 1	3.02 (2.34–3.90)	< 0.001	2.03 (1.61–2.57)	< 0.001
Model 2	2.94 (2.27–3.81)	< 0.001	1.84 (1.45–2.34)	< 0.001
Model 3	2.31 (1.73–3.08)	< 0.001	1.59 (1.25–2.04)	< 0.001

Model 1: unadjusted; Model 2: age, sex, and body mass index; Model 3: Model 2 plus hypertension, diabetes, estimated glomerular filtration rate, urinary protein, and urinary red blood cell count. Abbreviations: CI, confidence interval; FENa, fractional excretion of sodium; HR, hazard ratio; PSI, proteinuria selectivity index.

Supplementary Table 4. Results of Cox regression analyses for complete remission (only patients who received immunosuppressive therapy)

	PSI		FENa	
	HR (95%CI)	P value	HR (95%CI)	P value
Model 1	3.68 (2.70–5.01)	< 0.001	2.35 (1.78–3.10)	< 0.001
Model 2	3.77 (2.74–5.18)	< 0.001	2.14 (1.61–2.85)	< 0.001
Model 3	2.89 (2.05–4.09)	< 0.001	1.97 (1.47–2.64)	< 0.001

Model 1: unadjusted; Model 2: age, sex, and body mass index; Model 3: Model 2 plus hypertension, diabetes, estimated glomerular filtration rate, urinary protein, and urinary red blood cell count. Abbreviations: CI, confidence interval; FENa, fractional excretion of sodium; HR, hazard ratio; PSI, proteinuria selectivity index.

Supplementary Table 5. Results of Cox regression analyses for complete remission (subdistribution hazard model)

	PSI		FENa	
	HR (95%CI)	P value	HR (95%CI)	P value
Model 1	3.83 (2.88–5.08)	< 0.001	2.42 (1.86–3.14)	< 0.001
Model 2	3.69 (2.74–4.95)	< 0.001	2.18 (1.67–2.86)	< 0.001
Model 3	2.83 (2.02–3.96)	< 0.001	1.93 (1.47–2.54)	< 0.001

Model 1: unadjusted; Model 2: age, sex, and body mass index; Model 3: Model 2 plus hypertension, diabetes, estimated glomerular filtration rate, urinary protein, and urinary red blood cell count. Abbreviations: CI, confidence interval; FENa, fractional excretion of sodium; HR, hazard ratio; PSI, proteinuria selectivity index.

Supplementary Table 6. Results of Cox regression analyses for complete remission (multiple imputations for missing variables)

	PSI		FENa	
	HR (95%CI)	P value	HR (95%CI)	P value
Model 1	3.61 (2.71–4.83)	< 0.001	2.37 (1.82–3.10)	< 0.001
Model 2	3.50 (2.61–4.69)	< 0.001	2.11 (1.61–2.77)	< 0.001
Model 3	2.86 (2.06–3.98)	< 0.001	1.86 (1.41–2.46)	< 0.001

Model 1: unadjusted; Model 2: age, sex, and body mass index; Model 3: Model 2 plus hypertension, diabetes, estimated glomerular filtration rate, urinary protein, and urinary red blood cell count. Abbreviations: CI, confidence interval; FENa, fractional excretion of sodium; HR, hazard ratio; PSI, proteinuria selectivity index.
